# Analysis of potential regulatory LncRNAs and CircRNAs in the oxidative myofiber and glycolytic myofiber of chickens

**DOI:** 10.1038/s41598-021-00176-y

**Published:** 2021-10-21

**Authors:** Xiaojun Ju, Yifan Liu, Yanju Shan, Gaige Ji, Ming Zhang, Yunjie Tu, Jianmin Zou, Xingyong Chen, Zhaoyu Geng, Jingting Shu

**Affiliations:** 1 College of Animal Science and Technology, Anhui Agricultural University, Hefei, 230036 Anhui China; 2 Key Laboratory for Poultry Genetics and Breeding of Jiangsu Province, Poultry Institute, Chinese Academy of Agricultural Sciences, Yangzhou, 225125 Jiangsu China

**Keywords:** Genetics, Molecular biology, Zoology

## Abstract

SART and PMM are mainly composed of oxidative myofibers and glycolytic myofibers, respectively, and myofiber types profoundly influence postnatal muscle growth and meat quality. SART and PMM are composed of lncRNAs and circRNAs that participate in myofiber type regulation. To elucidate the regulatory mechanism of myofiber type, lncRNA and circRNA sequencing was used to systematically compare the transcriptomes of the SART and PMM of Chinese female Qingyuan partridge chickens at their marketing age. The luminance value (L*), redness value (a*), average diameter, cross-sectional area, and density difference between the PMM and SART were significant (*p* < 0.05). ATPase staining results showed that PMMs were all darkly stained and belonged to the glycolytic type, and the proportion of oxidative myofibers in SART was 81.7%. A total of 5 420 lncRNAs were identified, of which 365 were differentially expressed in the SART compared with the PMM (*p* < 0.05). The cis-regulatory analysis identified target genes that were enriched for specific GO terms and KEGG pathways (*p* < 0.05), including striated muscle cell differentiation, regulation of cell proliferation, regulation of muscle cell differentiation, myoblast differentiation, regulation of myoblast differentiation, and MAPK signaling pathway. Pathways and coexpression network analyses suggested that XR_003077811.1, XR_003072304.1, XR_001465942.2, XR_001465741.2, XR_001470487.1, XR_003077673.1 and XR_003074785.1 played important roles in regulating oxidative myofibers by TBX3, QKI, MYBPC1, CALM2, and PPARGC1A expression. A total of 10 487 circRNAs were identified, of which 305 circRNAs were differentially expressed in the SART compared with the PMM (*p* < 0.05). Functional enrichment analysis showed that differentially expressed circRNAs were involved in host gene expression and were enriched in the AMPK, calcium signaling pathway, FoxO signaling pathway, p53 signaling pathway, and cellular senescence. Novel_circ_004282 and novel_circ_002121 played important roles in regulating oxidative myofibers by PPP3CA and NFATC1 expression. Using lncRNA-miRNA/circRNA-miRNA integrated analysis, we identified many candidate interaction networks that might affect muscle fiber performance. Important lncRNA-miRNA-mRNA networks, such as lncRNA-XR_003074785.1/miR-193-3p/PPARGC1A, regulate oxidative myofibers. This study reveals that lncXR_003077811.1, lncXR_003072304.1, lncXR_001465942.2, lncXR_001465741.2, lncXR_001470487.1, lncXR_003077673.1, XR_003074785.1, novel_circ_004282 and novel_circ_002121 might regulate oxidative myofibers. The lncRNA-XR_003074785.1/miR-193-3p/PPARGC1A pathway might regulate oxidative myofibers. All these findings provide rich resources for further in-depth research on the regulatory mechanism of lncRNAs and circRNAs in myofibers.

## Introduction

Myofiber is the basic constituent unit of skeletal muscle. Different types of myofibers give skeletal muscle-specific physiological characteristics and functions^1,2^. It is generally believed that the total number of myofibers remains unchanged after birth. However, these myofibers are dynamic structures capable of changing their phenotype during the growth process^3^. In chickens, myofibers can be divided into oxidative (type I and IIa, red) and glycolytic (type IIb, white) types^4^. The meat quality of muscle with a high proportion of oxidative myofibers is significantly better than that of muscle with a high proportion of glycolytic myofibres^5^. Compared to glycolytic myofibers, oxidative myofibers are characterized by a higher content of mitochondria, lipids, and myoglobin and a smaller diameter^6^. Previous studies have found that oxidative myofibers improved muscle growth and meat quality by affecting meat pH^7–9^, meat color^10–12^, and water-holding capacity^8,13^ in postnatal chickens. A higher content of oxidative myofiber makes the meat ruddy, fresh and juicy, which improves meat flavor, while a higher content of glycolytic myofiber makes the meat white and leads to reduced quality^14^. Therefore, how to regulate myofibers into the oxidative type is an important way to improve meat quality^2,15–17^.

Recent studies, most of which have been conducted among mice and pigs, have revealed the molecular mechanisms and possible signaling pathways involved in the proliferation of myoblasts and myofiber type. The expression level of PPAR in oxidative myofibers is significantly higher than that in glycolytic myofibers, and PPAR can induce the transformation of the oxidative type to glycolytic myofibers in mice^18^. Knocking out the AMPKβ2 gene could reduce the proportion of glycolytic myofibers in mice, and the AMPKβ2 gene plays an important role in the specificity and transformation of myofibers^19^. PPARGC1a is a major regulatory factor of mitochondrial synthesis and metabolism. In a mouse model, PPARGC1a cooperates with the MEF2 protein to activate the expression of oxidative fibro-related genes and is involved in the CaN signaling pathway as the target gene^20^. Overexpression of the PPARGC1a gene could promote glycolytic myofiber transformation to oxidative myofibers in pigs^21^. Deletion of the MSTN gene induces muscle hypertrophy and increases the formation of glycolytic myofiber^22^. The role of noncoding RNAs (ncRNAs), especially miRNAs, in myofiber regulation has been extensively studied^23^. Several recent studies have also shown that long noncoding RNAs (lncRNAs) and circular RNAs (circRNAs) might also be involved in the regulation of myofiber type^24–26^. Currently, the in-depth exploration of lncRNAs and circRNAs involved in myofiber type is a new direction for meat quality improvement research. Shen et al.^27^ used RNA-seq to discover a mass of candidate lncRNAs and circRNAs involved in porcine muscle physiological functions, which improved the understanding of muscle metabolism and development from an epigenetic perspective. Cai et al.^28^ found a novel muscle atrophy-associated lncRNA named SMUL that participates in the regulation of the transforming growth factor β (TGF-β)/SMAD pathway and further regulates myogenesis and muscle atrophy. Wang et al.^29^ found that lncRNA DLEU2 acts as a miR-181a sponge to regulate SEPP1 and inhibit skeletal muscle differentiation and regeneration. Gong et al.^30^ found that a long noncoding RNA, LncMyoD, regulates skeletal muscle differentiation by blocking IMP2-mediated mRNA translation. Legnini et al.^31^ found that circ-ZNF609 precisely controlled myoblast proliferation. Ouyang et al.^32^ found that circRBFOX2 can sponge miR-206 and negatively regulate miR-206 expression, thus increasing CCND2 expression and promoting myoblast proliferation. Peng et al.^33^ found that circSNX29 acts as a miR-744 sponge and that increased Wnt5a and CaMKIId expression results in the activation of noncanonical Wnt pathways and myoblast differentiation. Currently, only a few lncRNAs and circRNAs have been identified for myofiber type determination in chickens.

Qingyuan partridge chicken, an important indigenous breed in China, is popular because of its high red muscle ratio, appealing meat color and flavor^34,35^. The breed’s high oxidative metabolism leads to desirable muscle characteristics suitable for the study of muscle fiber types. In this study, the differential expression of lncRNAs and circRNAs between glycolytic muscle pectoralis major (PMM) and oxidative muscle sartorius (SART) of Chinese Qingyuan partridge chickens was analyzed to investigate the internal regulatory factors that regulate myofiber type.

## Results and discussion

### Phenotypic differences traits in oxidative and glycolytic myofiber

**Figure 1:**
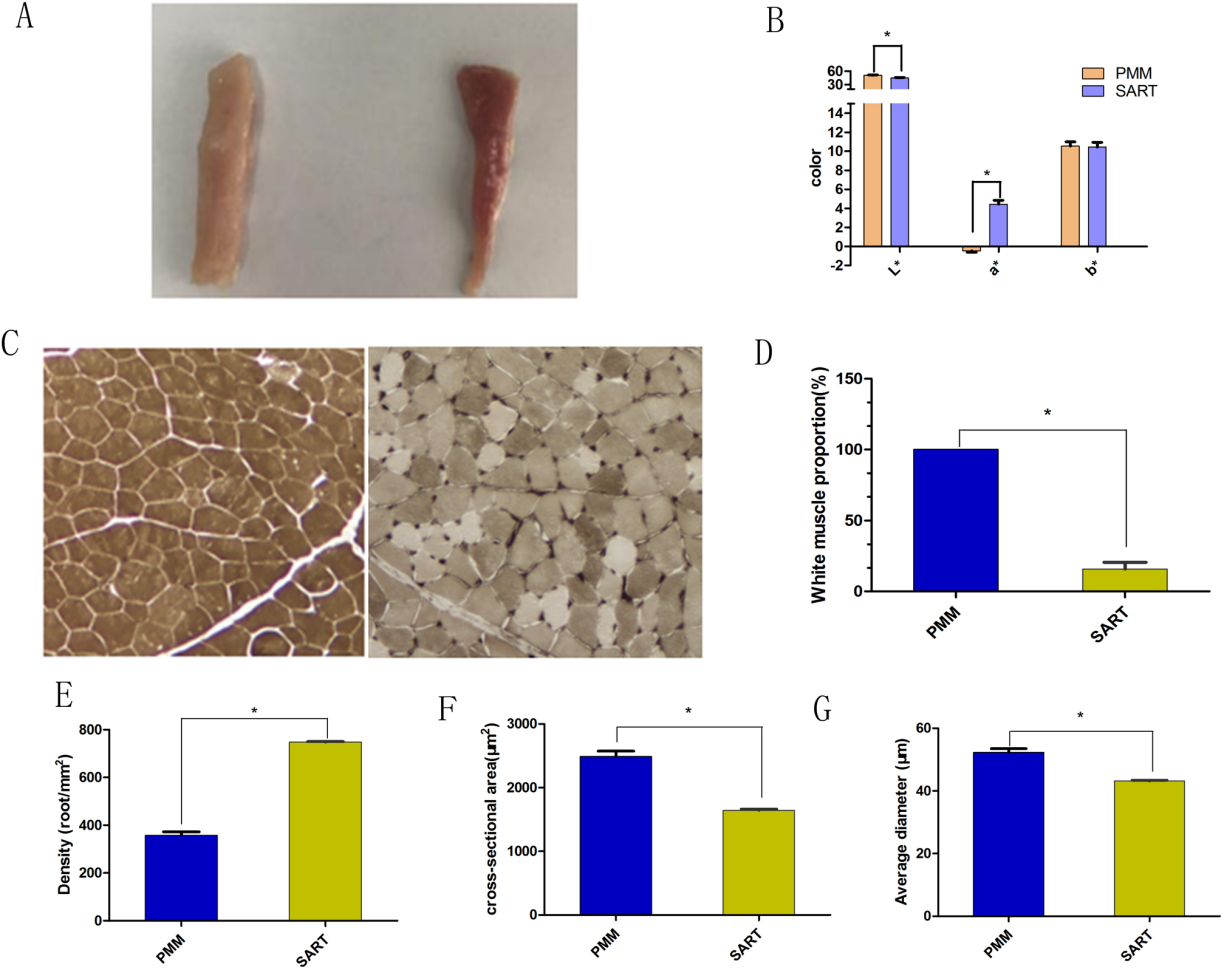
Different phenotypic indices between (pectoralis major) PMM and (sartorius major) SART muscles. (**A**) Fresh samples of PMM and SART, n = 15. Left: PMM, right: SART; (**B**) The color of fresh samples of PMM and SART, n = 15; (**C**) ATPase alkaline incubation staining of muscles in PMM and SART, n = 15. Left: PMM, right: SART; (**D**) The myofiber type ratio (white/red) between PMM and SART, n = 15; (**E**–**G**) The cross-sectional area, density, and average diameter between PMM and SART, n = 15. Data are means ± SEM. Statistical significance was calculated by Student’s t-test. Significant difference levels: *p < 0.05.

The luminance value (L*) of PMM was significantly higher than that of SART (*p* < 0.05), and the redness value (a*) of PMM was significantly lower than that of SART (*p* < 0.05, Fig. 1A,B). The PMMs were all darkly stained and belonged to the glycolytic type, and the proportion of oxidative myofibers in sartorius muscle was 81.7%, indicating that sartorius muscle was mainly composed of oxidative myofibers (Fig. 1C,D). The average diameter and cross-sectional area of the PMM were significantly higher than those of the SART (*p* < 0.05, Fig. 1E–G), and the myofiber density was significantly higher than that of the SART (*p* < 0.05). The PMM and SART muscle could be used as ideal models to study myofiber types.

### Screening of mRNAs, lncRNAs, and cricRNAs in oxidative and glycolytic myofibers

**Figure 2:**
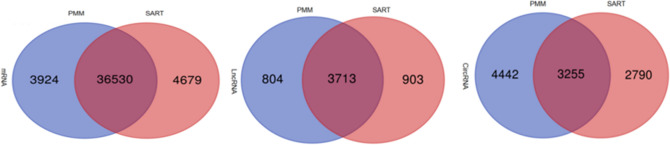
Venn diagrams represent all the numbers of expressed mRNAs, lncRNAs, and circRNAs between PMM and SART.

The RNA-Seq data from 8 cDNA libraries obtained 683,535,340 raw reads and 676,264,578 clean reads (Table 1). After discarding adaptor sequences and low-quality reads, approximately 78.50–85.48% of all reads were uniquely mapped to the chicken chromosomes, 0.59 to 1.04% were multiple mapped reads to the chicken chromosomes, and 79.27 to 86.13% of the clean reads were aligned against the Gallus gallus reference genome (Table 2). A total of 40,454 and 41,209 mRNAs were specifically expressed in the PMM and SART muscles, respectively, and 36,530 were expressed in both types of muscles. A total of 4517 and 4616 lncRNAs were specifically expressed in PMM and SART muscles, respectively, and 3713 were expressed in both types of muscles. A total of 7697 and 6045 circRNAs were specifically expressed in PMM and SART muscles, respectively, and 3255 were expressed in both types of muscles (Fig. 2).

**Table 1:** RNA-seq date quality evaluation.

Sample	Raw reads num	Clean reads num	Raw data (Gb)	Clean data (Gb)	Clean DataQ20 (%)	GC (%)	rRNA reads (%)
PMM1	86,283,566	85,474,788	12.94	12.67	98.58	48.11	0.27
PMM2	80,733,792	79,807,686	12.11	11.82	98.61	48.36	0.34
PMM3	87,308,752	86,360,624	13.10	12.79	98.54	47.86	0.28
PMM4	81,087,082	80,203,290	12.16	11.88	98.76	49.04	0.53
SART1	80,773,526	79,978,602	12.12	11.86	98.66	46.74	0.62
SART2	94,089,360	93,159,004	14.11	13.82	98.60	46.99	0.93
SART3	82,498,530	81,695,656	12.37	12.11	98.68	46.85	0.72
SART4	90,760,732	89,584,928	13.61	13.26	98.58	46.93	0.73

**Table 2:** The results of mapped reads.

Sample	Total reads	Unmapped reads (%)	Unique mapped reads (%)	Multiple mapped reads (%)	Mapping ratio (%)
PMM1	85,243,206	16.57	82.66	0.77	83.43
PMM2	79,534,030	16.35	82.61	1.04	83.65
PMM3	86,120,530	15.48	83.58	0.94	84.52
PMM4	79,780,262	20.73	78.50	0.77	79.27
SART1	79,486,402	14.71	84.61	0.68	85.29
SART2	92,293,538	16.58	82.83	0.59	83.42
SART3	81,106,154	13.87	85.48	0.66	86.13
SART4	88,934,220	14.87	84.41	0.72	85.13

### Identification of differentially expressed mRNAs, lncRNAs, and cricRNAs

**Figure 3:**
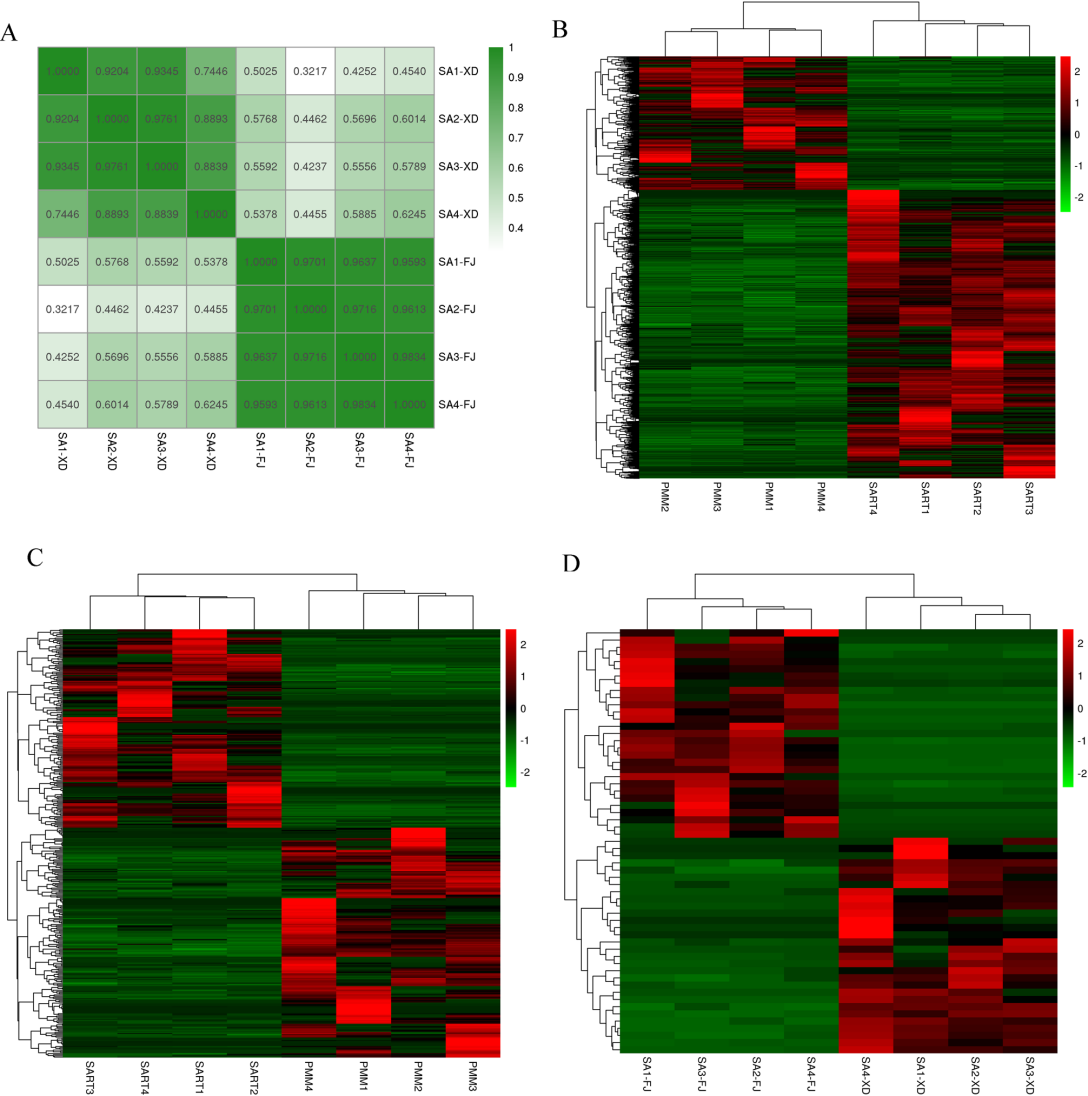
Heatmaps of sample correlation and differentially expressed RNAs between PMM and SART. (**A**) Sample consistency. Dark green indicates a high correlation. (**B**) Heatmap for DE-mRNA. (**C**) Heatmap for DE-lncRNA. (**D**) Heatmap for DE-circRNA.

In this study, replicates were highly correlated, with an average correlation coefficient of 0.92 (Fig. 3A). There were 3457 differentially expressed mRNAs (DE-mRNAs), including 2364 upregulated and 1093 downregulated mRNAs in the SART compared to the PMM. There were 365 differentially expressed lncRNAs (DE-lncRNAs), including 169 upregulated and 196 downregulated lncRNAs (Supplementary Table S1). There were 305 differentially expressed circRNAs (DE-circRNAs), including 111 upregulated and 194 downregulated circRNAs (Supplementary Table S2). Heatmaps generated from the expression of DE-mRNA, DE-lncRNA, and DE-circRNA were used to show the expression patterns of these RNAs between PMM and SART (Fig. 3B–D). These results proved the high reproducibility and reliability of transcriptome profiling performed in the present study.

### Functional enrichment analysis of neighboring target genes of differentially expressed lncRNAs

**Figure 4:**
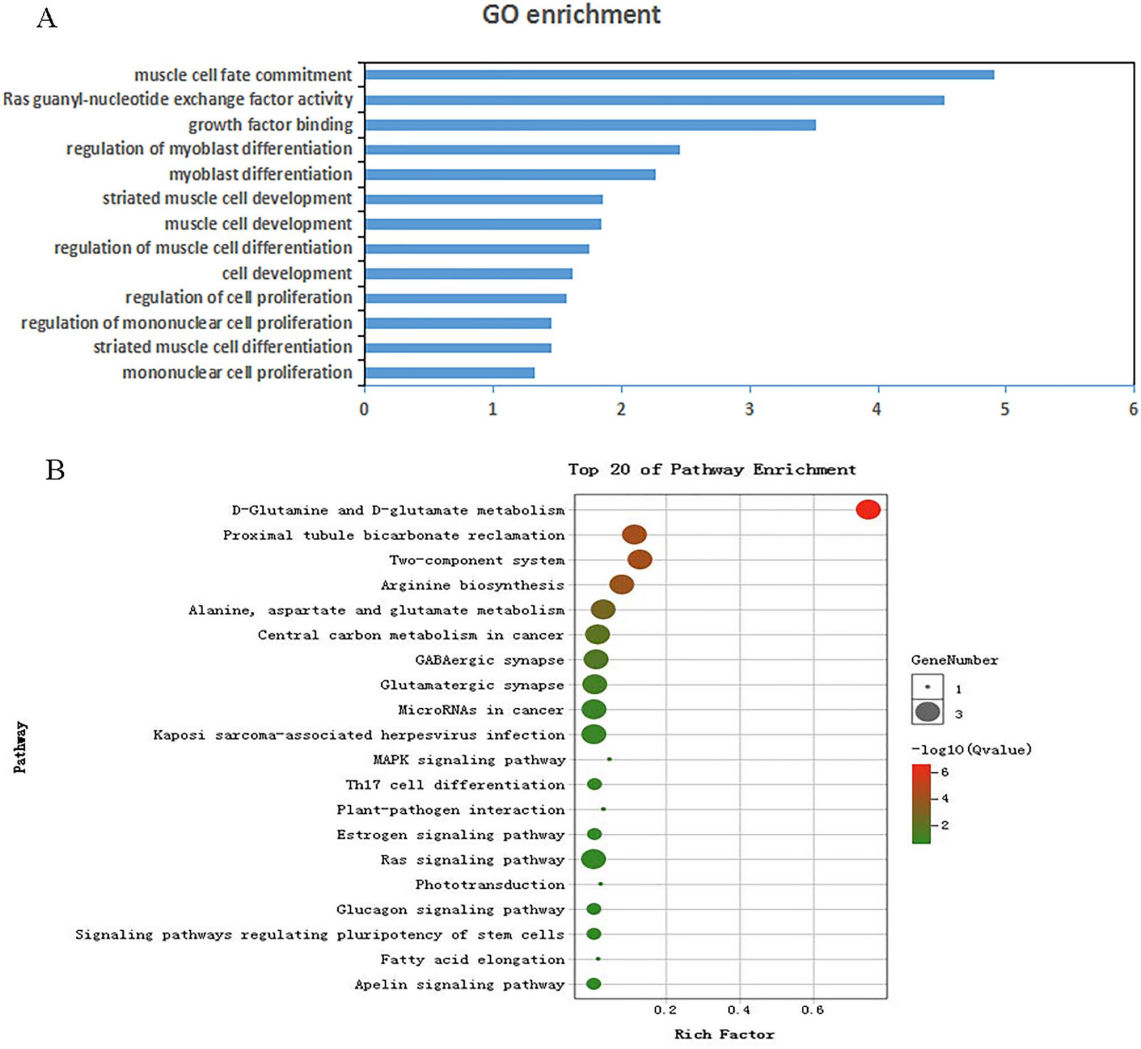
Functional enrichment analysis of neighboring target genes of DE-lncRNAs between PMM and SART. (**A**) Gene Ontology (GO) terms and (**B**) KEGG pathways enriched for neighboring target genes of DE-lncRNAs (cis-regulation)^43–45^.

Recent studies suggested that lncRNAs may act in cis-regulation and affect the gene expression of their chromosomal neighborhood 10 kb upstream and downstream^36,37^. In this study, 49 significantly differentially expressed lncRNAs in PMM vs. SART were transcribed close to (< 10 kb) 70 mRNAs. Gene Ontology (GO) analysis of the cis lncRNA target genes was performed to explore their functions (GO, https://www.omicshare.com/tools/home/report/goenrich.html) (Fig. 4A). There were 178 extremely significantly enriched GO terms (*p* < 0.05) in PMM vs. SART. Many of the significantly enriched biological processes were associated with muscle cell differentiation and myofiber regulation, such as striated muscle cell differentiation, regulation of cell proliferation, regulation of muscle cell differentiation, myoblast differentiation, and regulation of myoblast differentiation. Interestingly, T-box 3 (TBX3) was annotated in many myoblast differentiation-related GO terms. T-box3 (TBX3) could regulate the cell cycle, inhibit cell apoptosis and promote proliferation^38,39^ and was regulated by XR_003077811.1 in this study, which suggested that lncRNAs played important roles in regulating myofiber type.

To further understand the DE-lncRNA regulatory roles in myofiber type, Kyoto Encyclopedia of Genes and Genomes (KEGG, https://www.omicshare.com/tools/home/report/koenrich.html) pathway analysis was performed according to the cis-target genes (Fig. 4B). The MAPK signaling pathway was identified as a significantly enriched pathway for oxidative and glycolytic myofibers. Additionally, the MAPK family plays crucial roles in complex cellular processes, such as proliferation, differentiation, and development, by regulating the cell cycle and other cell proliferation-associated proteins^40,41^. Interestingly, only CALM2 was found to be differentially expressed in the MAPK signaling pathway and was regulated by XR_001465741.2. CALM2, as one subunit of CaM^42^, is a key redox-sensitive modulator of muscle physiology. Therefore, XR_001465741.2 was speculated to play a key role in myofiber growth.

### Functional enrichment analysis of host genes targeted by differentially expressed circRNAs

**Figure 5:**
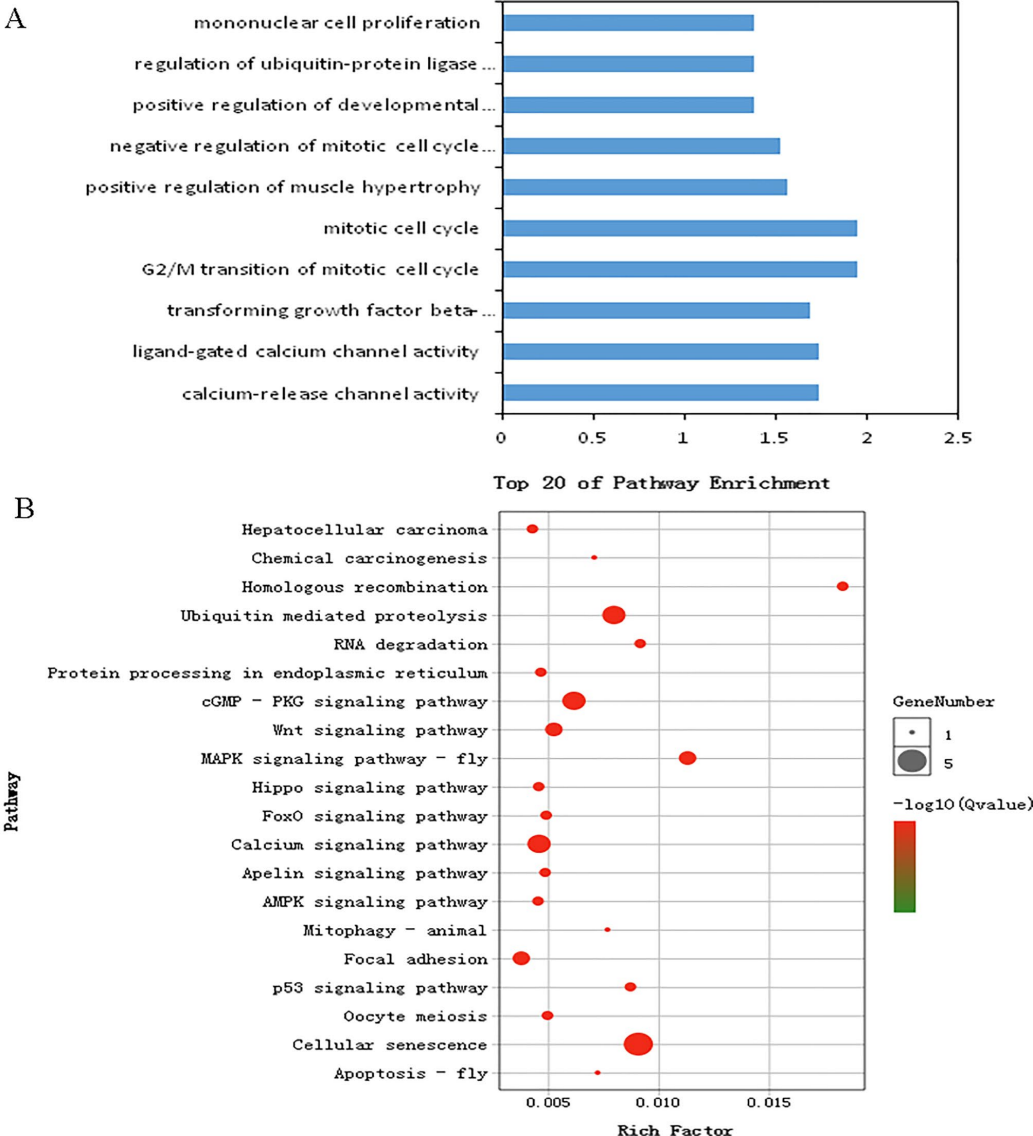
Functional enrichment analysis of differentially expressed host genes of circRNA between PMM and SART. (**A**) Gene Ontology (GO) terms and (**B**) KEGG pathways enriched for host genes of DE-circRNAs^43–45^.

Many studies have shown that circRNA expression can promote the transcription of host genes^46–48^. Therefore, to explore the mechanism of DE-circRNAs in myofibers, the host genes of DE-circRNA were used to perform a functional enrichment analysis (Fig. 5). There were 112 significantly enriched GO terms (*p* < 0.05) identified in the PMM and SART. The significantly overrepresented gene ontology (GO) terms were related to positive regulation of muscle hypertrophy, mitosis, positive regulation of developmental growth, and mononuclear cell proliferation (Fig. 5A). KEGG pathways, calcium signaling pathway, AMPK signaling pathway, FoxO signaling pathway, p53 signaling pathway, cellular senescence, etc., were closely related to myofiber development, resulting in the transformation of myofiber types (Fig. 5B). Ca^2+^ signaling pathways were implicated at every step of myogenesis^49^, which contributed to the transformation of glycolytic myofibers to oxidative myofiber type^50^. AMPK has three subunits: α, β and γ; α is the catalytic subunit; and β and γ are the regulatory subunits. The distribution of the three subunits was related to muscle fiber type, and the β2 subunit was highly expressed in skeletal muscle and mainly distributed in glycolytic muscle^51^. The AMPK signaling pathway regulates the differentiation directions of myoblasts and changes the types of myofiber^52^. The FoxO signaling pathway is regulated by a variety of phosphorylated kinases and plays an important role in the proliferation and differentiation of myoblasts and the transformation of myofiber type^53^. p53-related genes promoted the cell cycle by upregulating p21 and enhancing muscle differentiation in MSTN knockout QM7 cells^54^. In this study, the host genes CAB39L, FOXO3, BMPR1B, SESN1, UBE2D1, PPP1CB, XPO1, NFATC1, BMPR1B, and PPP3CA of novel_circ_001395, novel_circ_003490, novel_circ_004266, novel_circ_003485, novel_circ_005184, novel_circ_003035, novel_circ_002810, novel_circ_002121, novel_circ_004266, and novel_circ_004282 were involved in these pathways. These results suggest that these circRNAs might play important roles in regulating myofiber type.

Interestingly, differentially expressed novel_circ_004282 and novel_circ_002121 were transcribed from the PPP3CA and NFATC1 genes, respectively, which are CaN substrates involved in maintaining and regulating muscle phenotypes, promoting the growth of different types of myofibers, and maintaining muscle mass in the skeletal muscle of mammals after birth^55–57^. The CaN-NFAT pathway was the best candidate for an activity-dependent signaling pathway responsible for the maintenance of the oxidative gene program in adult oxidative muscles and the induction of this program in regenerating oxidative muscle^58^. These results suggested that novel_circ_004282 and novel_circ_002121 might directly regulate fiber type heterogeneity in muscles.

### LncRNA-mRNA interactions

**Figure 6:**
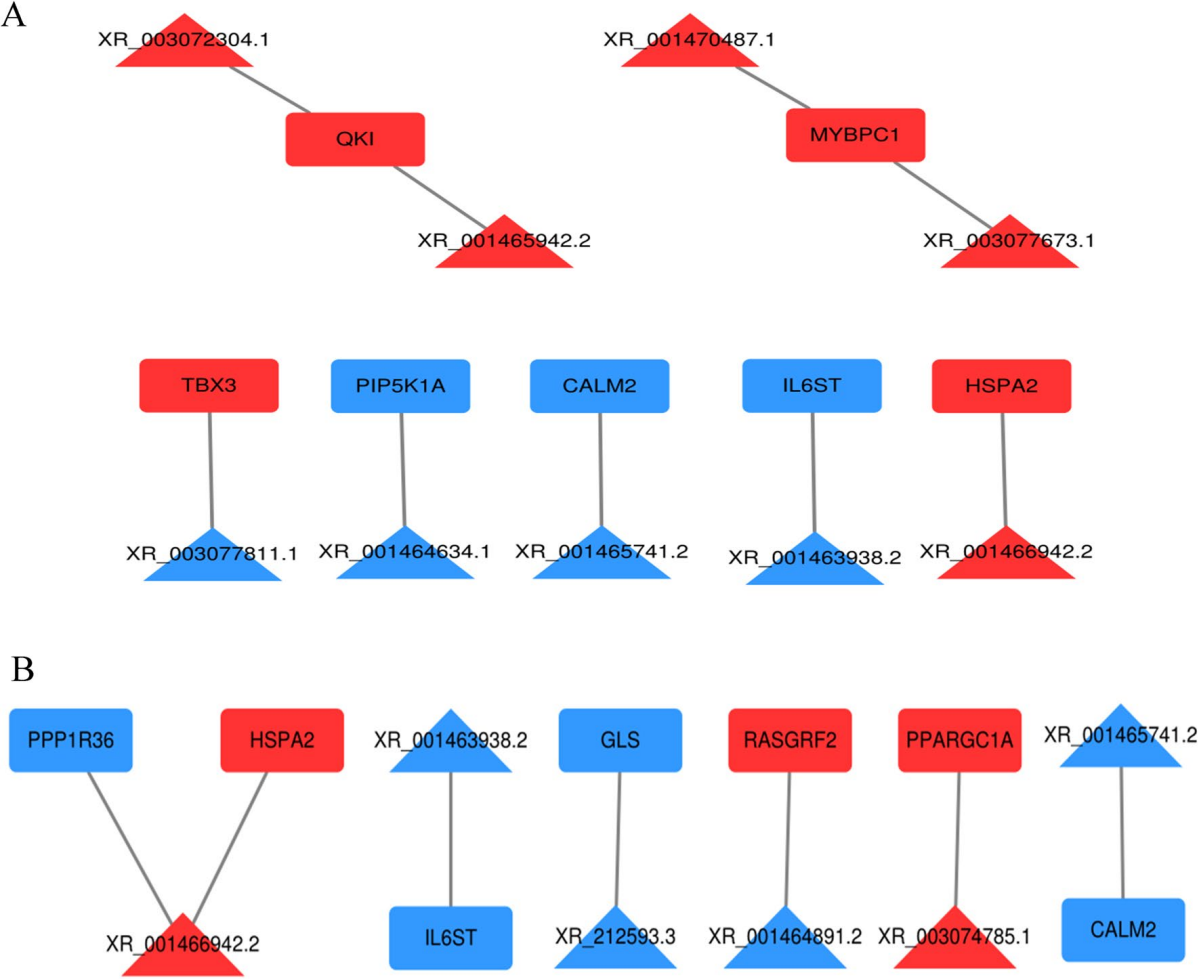
lncRNA-mRNA interactions for the selected cis-target DE genes. (**A**) lncRNA-mRNA interactions related to the GO terms. (**B**) lncRNA-mRNA interactions related to the KEGG signaling pathways. Genes are shown in rectangles, and lncRNAs are shown in triangles. RNA exhibiting upregulation is shown in red, whereas RNA exhibiting downregulation is shown in green.

To explore how lncRNAs interact with their target genes to regulate chicken myofibers and to identify key molecular players in the process, regulatory networks between DE-lncRNAs and their target DE genes were constructed. A total of 24 cis-regulatory interaction relationships were detected between 21 DE-lncRNAs and 24 DE-mRNAs (see Supplementary Fig. S1 online). DE-lncRNAs targeted 70 mRNAs, of which 24 mRNAs were differentially expressed and subjected to GO enrichment and KEGG pathway analysis. Six genes and 8 lncRNAs generated 8 interactions in GO terms (Fig. 6A), while 7 genes and 6 lncRNAs generated 7 interactions in KEGG pathways (Fig. 6B). The interaction networks containing TBX3, QKI, HSPA2, MYBPC1, CALM2, and PPARGC1A, which were regulated by XR_003077811.1, XR_001465942.2 or XR_003072304.1, XR_001470487.1 or XR_003077673.1, XR_001465741.2, XR_003074785.1, etc., lncRNAs, were all related to myofiber type. The interactions of XR_001466942.2-HSPA2 and XR_003072304.1-HSPA2 were both enriched in GO terms and KEGG pathways.

Over the past decades, QKI has important functions in neural progenitors, myelin formation, smooth muscle differentiation, and monocyte to macrophage differentiation^59–61^. QKI could promote the myoblast differentiation of C2C12 cells in mice^31^. HSPA2 is abundantly expressed in skeletal muscle^62^ and is induced by exercise-associated oxidative stress^63^. It prevents apoptosis by interacting with apoptosis-inducing factors (AIFs)^64^. MYBPC1 is known to encode the oxidative myofiber isoform of the major myosin-binding proteins in muscles^65–67^ and acts as an adaptor to connect the ATP consumer (myosin) and the regenerator (MM-CK)^68^. The higher expression level of the MYBPC1 gene could result in more IMF deposition in skeletal muscle by controlling the energy metabolism and homeostasis of oxidative myofiber^69^. MYBPC1 could contribute to better meat quality of red muscle and was more highly expressed in oxidative myofibers than in glycolytic myofiber^70^. PPARGC1A is a coactivator of transcription involved in several aspects of skeletal muscle physiology, such as mitochondrial biogenesis, glucose utilization, fatty acid oxidation, thermogenesis, gluconeogenesis and insulin signaling^71^. Overexpression of PPARGC1A in mice reveals oxidative myofiber dominance^20^, while PPARGC1A knockout mice exhibit glycolytic myofiber dominance^72^. PPARGC1A has been shown to directly coactivate different myocyte enhancer factor 2 (Mef2) proteins involved in the induction and maintenance of muscle differentiation; this was considered critically important for switching among myofiber types or for myofiber transition to oxidative myofibers^20^. Previous research also confirmed that PPARGC1A was a key gene involved in chicken myofiber type^70^. In this study, QKI, HSPA2, MYBPC1, and PPARGC1A were all highly expressed in SART. Therefore, these results suggest that XR_003077811.1, XR_003072304.1, XR_001465942.2, XR_001465741.2, XR_001470487.1, XR_003077673.1 and XR_003074785.1 play important roles in regulating oxidative myofiber by TBX3, QKI, MYBPC1, CALM2, and PPARGC1A expression.

### LncRNA-miRNA/circRNA-miRNA interaction networks in oxidative and glycolytic myofibers

In a previous study, the microRNA transcriptomes of SART and PMM of Chinese Qingyuan partridge chickens were compared^4^. Sixty-seven differentially expressed miRNAs were identified. To explore the lncRNA-miRNA/miRNA-miRNA interaction network, the target relationship between miRNAs and lncRNAs (Supplementary Table S3) and the target relationship between miRNAs and circRNAs during SART and PMM (Supplementary Table S4) were predicted. The role of miRNAs in myofiber type, such as gga-miR-499, gga-miR-1, gga-miR-196-5p, gga-miR-193a-3p, gga-miR-34a-5p, gga-miR-221-5p, and gga-miR-126-3p, has been extensively studied. MYH7B (oxidative myosin) is regulated by miR-499 and is responsible for muscle performance^73^. MiR-499-5p plays roles in the specification of myofiber identity, thereby promoting the NFATc1/MEF2C pathway and then activating a series of oxidative myofiber gene programs^74^. The miR-499/Fnip1/AMPK signaling pathway could serve as a mechanism to couple myofiber type and mitochondrial function^75^. MiR-1 plays an important role in myoblast differentiation, regeneration, angiogenesis regulation, proapoptosis, and oxidative stress control^76^. miR-1 expression could be regulated by IGF1 via the IGF1–AKT–FOXO3–miR-1 axis^77^. MiR-1 creates a positive regulatory feedback loop by targeting HDAC4 (histone deacetylase 4), a repressor of MEF2, which subsequently results in the upregulation of miR-1^78^. MiR-196-5p was predicted to suppress CALM1 and MYLK4 expression, and CALM1 and MYLK4 were vital in regulating myofiber type^79^. Gga-miR-221, gga-miR-34a, and gga-miR-126 are involved in muscle cell differentiation and energy metabolisms^80–82^. Gga-miR-193-3p inhibited PPARGC1A expression in chicken muscles. Gga-miR-129-3p has multiple target genes that are involved in the CaN/NFAT signaling pathway, suggesting its roles in chicken myofiber regulation through the CaN/NFAT signaling pathway^4^.

**Figure 7:**
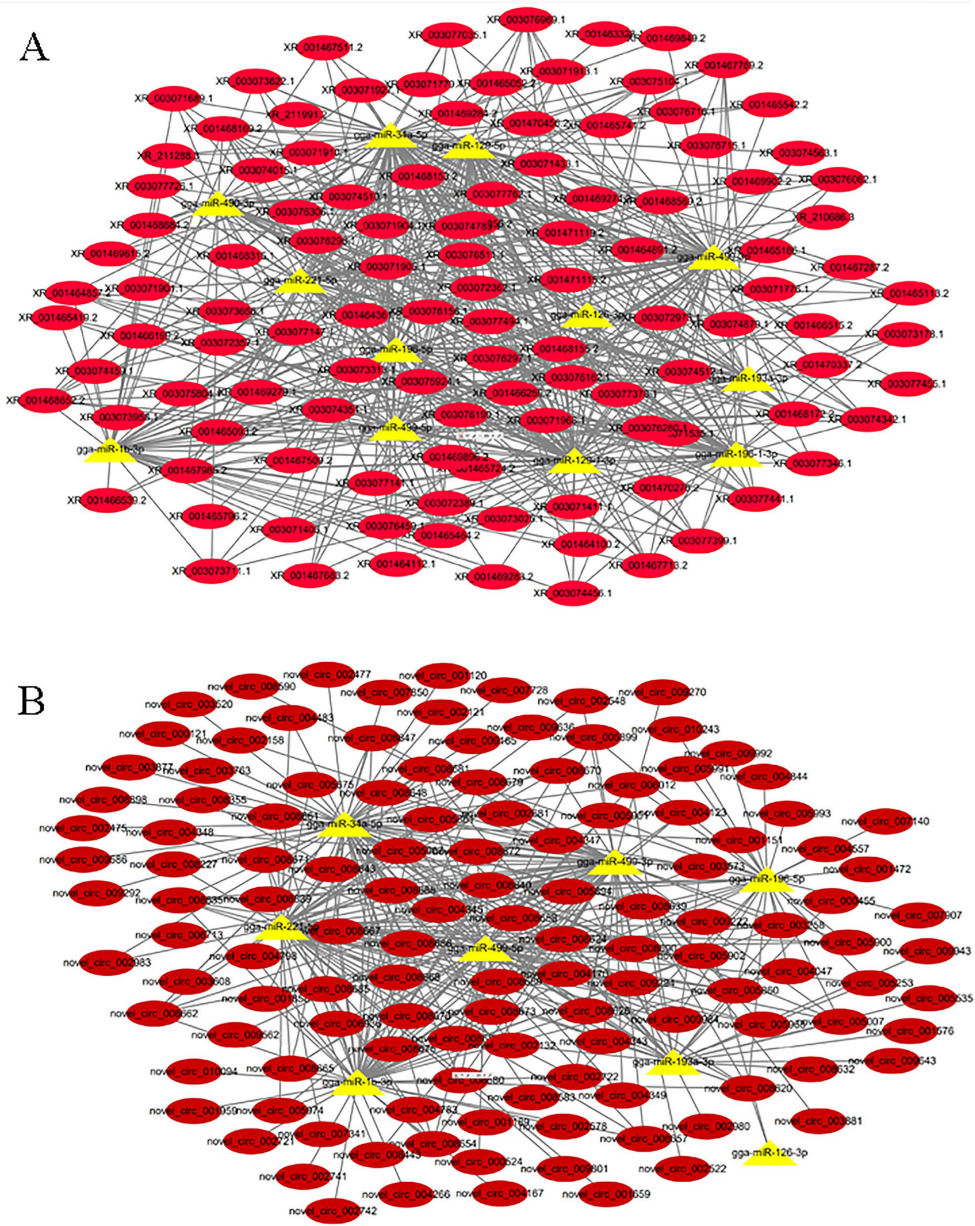
LncRNA-miRNA/circRNA-miRNA interaction network. (**A**) LncRNA-miRNA interaction network. LncRNA is shown in circular and red, miRNA is shown in the triangle, and yellow. (**B**) MircRNA-miRNA interaction network. CircRNA is shown in circular and red, miRNA is shown in the triangle, and yellow.

Therefore, gga-miR-499, gga-miR-1, gga-miR-196-5p, gga-miR-193a-3p, gga-miR-34a-5p, gga-miR-221-5p, and gga-miR-126-3p were selected as the targets to construct the miRNA-lncRNA interaction network diagram and miRNA-circRNA interaction network diagram (Fig. 7A,B). LncRNAs and circRNAs that regulate these miRNAs might regulate the type of myofiber.

### LncRNA-miRNA-mRNA regulatory networks

**Figure 8:**
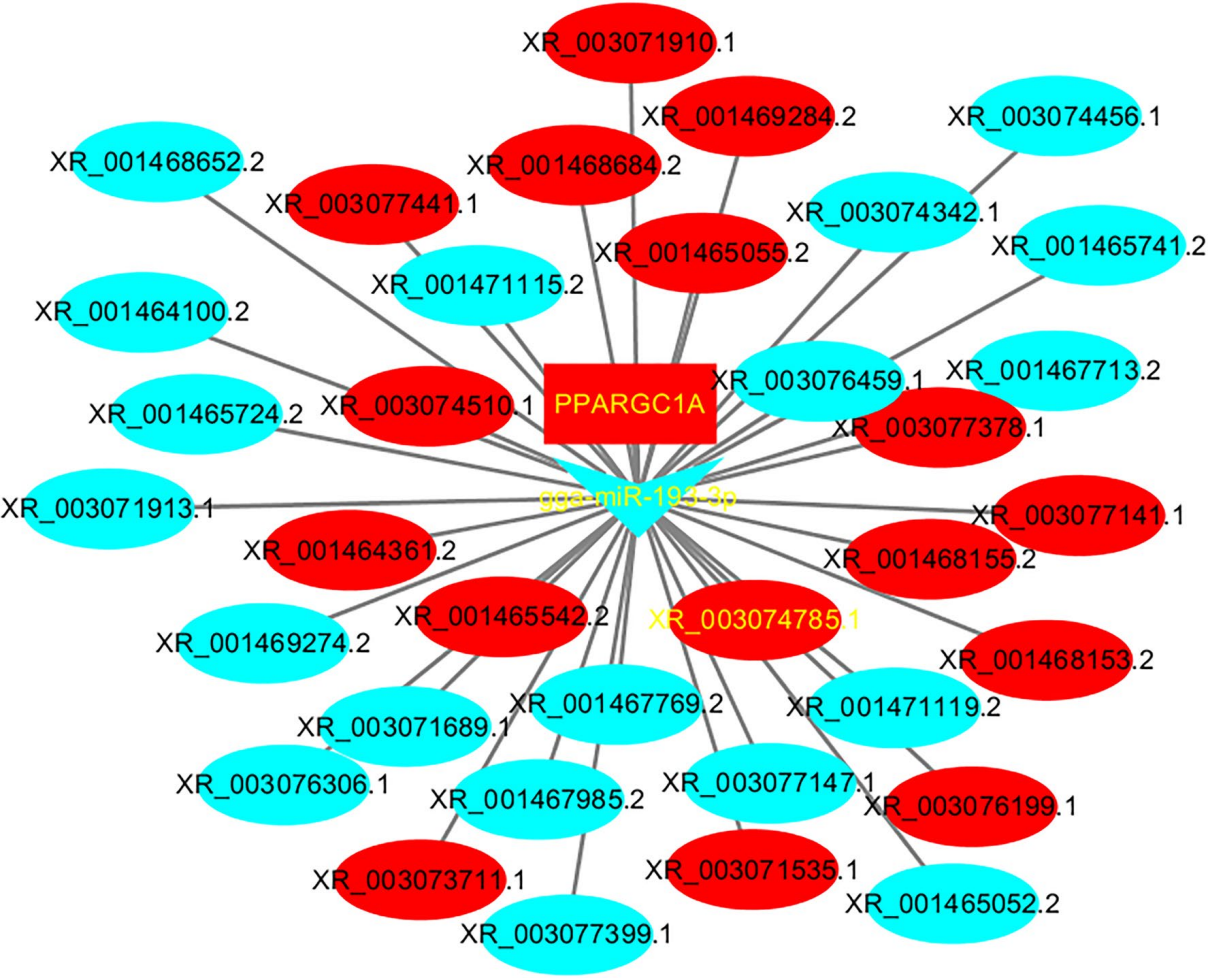
LncXR_003074785.1/miR-193-3p/PPARGC1A regulatory networks. LncRNA is shown in ellipse, miRNA is shown in the V, the gene is shown in the rectangle. Red is upregulated and blue is downregulated.

To identify potential lncRNA-miRNA-mRNA regulatory networks in oxidative myofibers and glycolytic myofibers, lncRNA-miRNA-mRNA regulatory networks of DE-genes, DE-miRNAs, and DE-lncRNAs were constructed. Interestingly, the lncRNA-miRNA-mRNA regulatory networks containing lncRNAs (XR_003074785.1) and its cis-target gene (PPARGC1A) might combine with gga-miR-193-3p, lncRNA (XR_003074785.1) and PPARGC1A were upregulated, and gga-miR-193-3p was downregulated. Therefore, XR_003074785.1 might competitively combine with miR-193-3p and then inhibit its combination with the PPARGC1A 3' UTR, which might regulate the myofiber type in chickens (Fig. 8).

### Validation of differentially expressed lncRNAs and circRNAs by qRT–PCR

**Figure 9:**
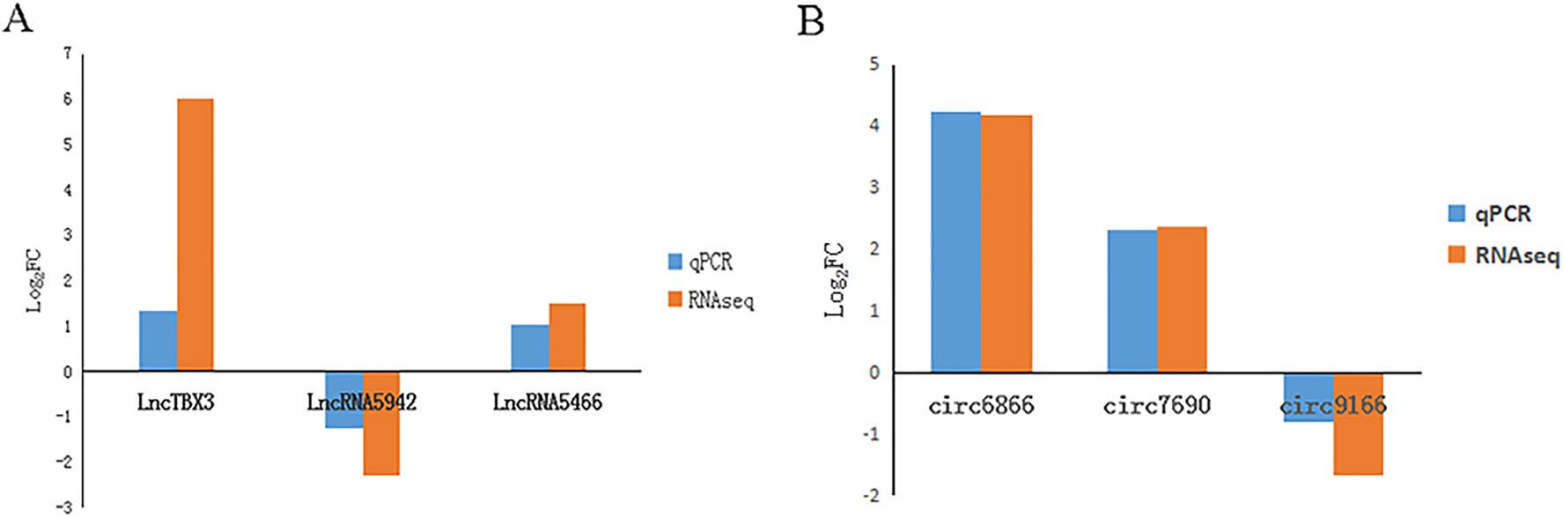
Using qRT–PCR to validate RNA-seq data. (**A**) RNA-seq and qRT–PCR data of the expression levels of lncRNAs, n = 3. (**B**) RNA-seq and qRT–PCR data of the expression levels of circRNAs, n = 3.

To validate the differential expression results of lncRNAs and circRNAs, the relative expression of three randomly selected lncRNAs and three randomly selected circRNAs was quantified by qRT–PCR. In Fig. 9A,B, all selected DE-lncRNAs and DE-circRNAs showed concordant expression patterns between the RNA-seq and qRT–PCR results.

## Materials and methods

### Ethics statement

All animal experiments were performed in accordance with the protocol of the Animal Use Committee of the Chinese Ministry of Agriculture and were approved by the Animal Care and Use Committee at the Poultry Institute, Chinese Academy of Agricultural Science. All experiments were performed in accordance with relevant guidelines and regulations. The animals were euthanized according to the American Veterinary Medical Association (AVMA) Guidelines for the Euthanasia of Animals (2020). All efforts were made to minimize animal suffering. The reporting in the manuscript follows the recommendations in the ARRIVE guidelines and was in accordance with relevant guidelines and regulations.

### Animal materials, tissue collection

A total of 15 female Qingyuan partridge chickens, provided by Guangdong Tiannong Food Ltd, Guangdong, China, with similar body weights were euthanized by stunning followed by exsanguination at 140 days of age (marketing age). Pectoralis major and sartorius major on the left side were sampled immediately after slaughter, one part of the samples was stored in liquid nitrogen for frozen sectioning, and the other part was stored in liquid nitrogen for RNA extraction. Pectoralis major and sartorius major on the right side were sampled and stored at 4 ℃ for meat color measurement.

### Meat color measurement

After 24 h of storage, meat color was measured by a color difference meter (Shenzhen 3NH Technology CO., Ltd, Guangdong, China). The average of triplicate measurements was recorded, and the results were expressed as lightness (L*) and redness (a*) values.

### Frozen section analysis

A 1 × 1 cm^2^ section in the middle of the right SART and PMM was selected. Measurement of the myofiber characteristics, including density, cross-sectional area, average diameter, and myofiber ratios, was carried out using ATPase staining. Myosin ATPase staining was used to identify myofiber type and to measure myofiber size^83,84^. The lightly dyed fibers were type I, the darker dyed fibers were type IIB, and the middle of the dyeing was type IIA. Oxidative myofibers included type I and IIA myofibers, and glycolytic myofibers were type IIB myofibers.

### RNA extraction

Total RNA was extracted by using TRIzol reagent (Invitrogen, CA, USA) following the manufacturer's protocol. The RNA quantity of each sample was examined using a NanoDrop ND-2000 spectrophotometer (Thermo Scientific, Waltham, MA, USA) at 260/280 nm (ratio > 2.0). The integrity of total RNA was analyzed with the Agilent Bioanalyzer 2100 and RNA 6000 Nano LabChip Kit (Agilent Technologies) with RNA integrity number (RIN) ≥ 7.

### Transcriptome library construction and sequencing

A total of eight cDNA libraries were constructed with four PMMs and four SART muscle tissues. A total of 3 µg RNA per sample was used as input material for RNA sample preparation. After the mRNAs and noncoding RNAs were enriched by removing rRNAs from the total RNA, the enriched RNAs were fragmented into short fragments and reverse transcribed into cDNAs. Buffer, dNTPs, RNase H, and DNA polymerase I were added to synthesize the second-strand cDNA. The resulting double-stranded cDNAs were ligated to adaptors after being end-repaired and A-tailed. Then, uracil-N-glycosylase (UNG) was used to digest the second-strand cDNAs. The digested products were size selected by agarose gel electrophoresis, PCR amplified, and sequenced by Gene Denovo Biotechnology Co. (Guangzhou, China) using Illumina HiSeq 4000^85^.

### Identification of lncRNAs and circRNA

The Illumina sequencing raw reads were obtained by removing adapter sequences, reads with poly-N, and low-quality reads, in which the number of bases with a quality value Q ≤ 20 was > 50%. All downstream analyses were based on high-quality clean data. The clean reads of each sample were mapped to the reference genome (Galgal 6.0) using TopHat (version 2.1.1)^86^. Reference genome and gene model annotation files were downloaded from a genome website (fttp://ftp.ensembl.org/pub/release-83/fasta/gallus_gallus/dna/). The mapped reads from each library were assembled with Cufflinks (version 2.1.1) to construct and identify mRNA transcripts^87^. Transcript abundance was quantified by RSEM software^88^.

Next, new transcripts were screened based on the position of the assembled transcript on the reference genome and the following screening criteria: transcript length ≥ 200 bp and the number of exons ≥ 2 (plant sample ≥ 1) to obtain known and new transcripts from the sample. Next, the Coding-Non-Coding Index (CNCI)^89^ and Coding Potential Calculator (CPC)^90^ were used to remove potential protein-coding transcripts. In this study, the resulting transcripts with no protein-coding potential in the two software analyses resulted in the lncRNA dataset.

In this study, find_circ software was used to identify circRNAs. Briefly, the unmapped back-spliced junction reads (default 20 bp) were used to extend the anchor sequences by find_circ software with default parameters^91^. In addition, identified circRNAs that were expressed in at least two samples were used for further analysis.

The expression levels of mRNAs, lncRNAs, and circRNAs were normalized using the fragments per kilobase of transcript per million mapped reads (FPKM) method. Differentially expressed mRNAs, lncRNAs, and cricRNAs were filtered by edgeR software with parameters of p value < 0.05 and | log2(fold change) |≥ 1^92^.

### Functional enrichment analysis

Differentially expressed lncRNAs were selected for cis-target gene predictions and were integrated with differentially expressed gene data to improve the veracity of target prediction. In the present study, DE-mRNAs located ∼10 kb upstream and downstream of DE-lncRNAs were classified as cis-acting target genes; then, their functional roles were predicted as follows. The host genes of DE-circRNA were used to perform a functional enrichment analysis.

All DE-mRNAs and target genes of DE-lncRNAs and DE-circRNAs were annotated and classified by Gene Ontology (GO) and Kyoto Encyclopedia of Genes and Genomes (KEGG) pathway analysis with OmicShare tools (HTTP ://www.omics hare.com/tools/)^43–45^. The results with a *p* value < 0.05 were considered significantly enriched. DE-mRNA and DE-lncRNAs were used to construct lncRNA-mRNA interaction networks by Cytoscape 3.5.1.

### Construction of lncRNA-miRNA-mRNA network

The lncRNA-miRNA-mRNA network was constructed based on lncRNA-miRNA-mRNA theory as follows: (1) Expression correlation between mRNA-miRNA or lncRNA-miRNA was evaluated using the Spearman rank correlation coefficient (SCC). Pairs with SCC < − 0.7 were selected as coexpressed negative lncRNA–miRNA pairs or mRNA-miRNA pairs, both mRNA and lncRNA were miRNA target genes, and all RNAs were differentially expressed. (2) The expression correlation between lncRNAs and mRNAs was evaluated using the Pearson correlation coefficient (PCC). Pairs with PCC > 0.9 were selected as coexpressed lncRNA–mRNA pairs, and both mRNA and lncRNA in this pair were targeted and coexpressed negatively with a common miRNA. (3) A hypergeometric cumulative distribution function test was used to test whether the common miRNA sponges between the two genes were significant. As a result, only the gene pairs with a p value less than 0.05 were selected^93–95^.

DE-miRNA, DE-mRNA, DE-lncRNAs, and DE-circRNAs were used to construct lncRNA-miRNA, circRNA-miRNA, and lncRNA-miRNA-mRNA interaction networks by Cytoscape 3.5.1.

### Validation by qRT–PCR

To validate the differential expression results from sequencing, three lncRNAs and three circRNAs were selected for qRT–PCR. Total RNA for sequencing was reverse transcribed into cDNA using the PrimeScript RT reagent kit (TaKaRa, Dalian, China). Then, qRT–PCR was conducted using a KAPA SYBR Fast universal qPCR kit (Kapa Biosystems, USA). Glyceraldehyde-3-phosphate dehydrogenase (GAPDH) genes were used as the internal reference. All primers are shown in Table 3.

**Table 3:** Primers for quantitative real-time PCR.

Gene symbol	Forward primer (5′–3′)	Reverse primer (5′–3′)
LncRNA5942	ATAGGGCAGGGGAGGCTAAATACC	GGTGTGTTGAGGCTCGTGATGG
LncRNA5466	CCACGGTTGACTTCAGCTCCTTC	TCCCGTAGTGTGTCTCCGATGTC
LncTBX3	TCCGCCAGTTCCGAGTGACC	AGACAAAGACTGTTCGCACCTTCC
Circ6866	TCGTGGCAGGAAGTCAGTGTC	TGAACATTCACCAAGGATCCGTGT
Circ7690	AACCAAAGCCTGGTCATACTCCT	GAGTGAGACTGCAGTCCATGGC
Circ9166	AACAGTGATGATTGGAGGAGAGCC	CAGAAAAGGAGTCTTTGGACAATGGT

### Statistical analysis

Comparisons of the two myofiber types were analyzed using an independent sample T-test procedure in SPSS (Version 20.0, SPSS, Inc., Chicago, IL, USA). Differences between PMM and SART muscle samples were considered statistically significant at *p* < 0.05.

## Supplementary Information

Supplementary Figure S1.

Supplementary Table S1.

Supplementary Table S2.

Supplementary Table S3.

Supplementary Table S4.

## Data Availability

The datasets supporting the results presented here are available in the Sequence Read Archive (SRA) repository under accession number PRJNA578179.
