# Supplementary material for: Analysis of potential regulatory LncRNAs and CircRNAs in the oxidative myofiber and glycolytic myofiber of chickens
Source: Sci Rep. 2021 Oct 21;11:20861. doi: 10.1038/s41598-021-00176-y (PMC8531282; doi:10.1038/s41598-021-00176-y)
Supplement: Supplementary file 1 — Supplementary Figure S1. [file 41598_2021_176_MOESM1_ESM.pdf]

Analysis of Potential Regulatory LncRNAs and CircRNAs in the Oxidative Myofiber and Glycolytic Myofiber of Chickens

Xiaojun Ju[1], Yifan Liu[2], Yanju Shan[2], Gaige Ji[2], Ming Zhang[2], Yunjie Tu[2], Jianmin Zou[2], Xingyong Chen[1], Zhaoyu Geng[1,*], Jingting Shu[2,*]

[1]College of Animal Science and Technology, Anhui Agricultural University, Hefei230036, Anhui, China.

[2]Key Laboratory for Poultry Genetics and Breeding of Jiangsu Province, Poultry Institute, Chinese Academy of Agricultural Sciences, Yangzhou 225125, Jiangsu, China.

Correspondence and requests for materials should be addressed to J.S or Z.G. (email: shujingting@163.com, gzy@ahau.edu.cn)

Supplementary Information

Supplementary Tables

Supplementary Table S1. Details of differentially expressed lncRNAs.

Supplementary Table S2. Details of differentially expressed circRNAs.

Supplementary Table S3. The interaction between DE-lncRNAs and DE-miRNAs.

Supplementary Table S4. The interaction between DE-circRNAs and DE-miRNAs.

Supplementary Figure

Supplementary Fig. S1

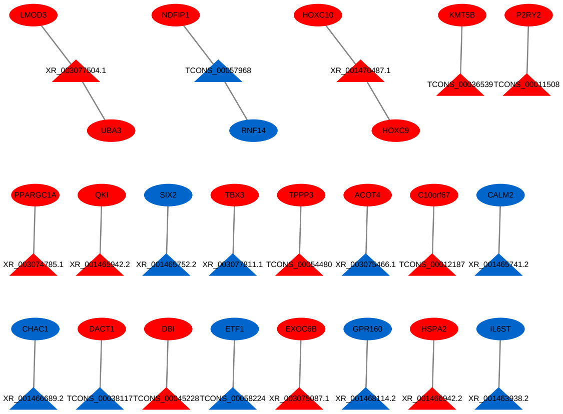

Supplementary Fig. S1. Interactions between DE-lncRNAs and DE-gene. Genes are shown in circular, lncRNA are shown in triangle.RNA exhibiting up-regulation are shown in red, whereas RNA exhibiting down-regulation are blue in green.
